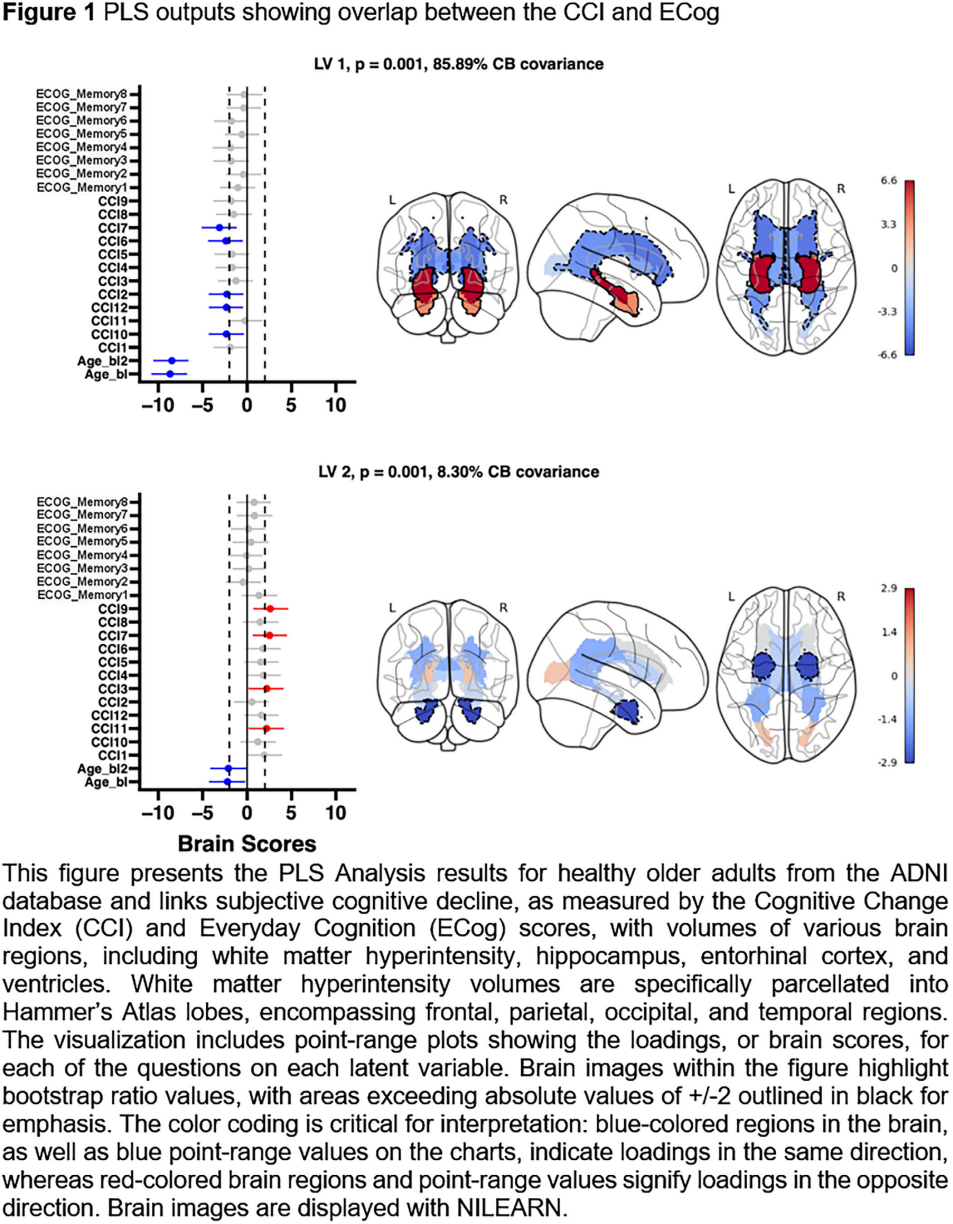# Neuroimaging Insights into Subjective Cognitive Decline: Unveiling Differential Sensitivity of Cognitive Change Index and Everyday Cognition Scale

**DOI:** 10.1002/alz.085932

**Published:** 2025-01-03

**Authors:** John Anderson, Mahsa Dadar, Cassandra Morrison

**Affiliations:** ^1^ Carleton University, Ottawa, ON Canada; ^2^ Douglas Mental Health University Institute, Montreal, QC Canada; ^3^ McGill University, Montreal, QC Canada

## Abstract

**Background:**

Subjective cognitive decline (SCD), or self‐perceived declines in memory/cognition in cognitively healthy older adults is linked to increased cognitive decline, neurodegeneration, and white matter hyperintensity (WMH) burden. However, there is no consistent definition of how to classify people with SCD. This study investigated if individual questions in the Cognitive Change Index (CCI) and Everyday Cognition Scale (ECog), commonly used to classify SCD, are associated with brain volume and WMHs to the same degree.

**Methods:**

A total of 332 cognitively healthy older adults from the Alzheimer’s Disease Neuroimaging Initiative were included in this study. Partial‐least‐squares was used to analyze the data and generate a set of orthogonal latent variables (LVs) that maximize the relationship between two sets of data. The significance of each LV was assessed by comparing the obtained result to a null distribution built with 1000 permutations. The reliability of the contributions of each of the variables to the LV was assessed with 1000 bootstrap repetitions, which were used to estimate standard errors.

**Results:**

Two significant LVs (*p*’s<0.001) explained 85.89% and 8.30% of the cross‐block covariance (Figure 1). The first LV shows higher SCD scores correlate with older age and fontal, parietal, and temporal WMH burden. This pattern also correlated with lower gray matter in the entorhinal cortex, and hippocampus and larger ventricles. These changes were associated with questions from only the CCI suggesting this questionnaire is more sensitive than the ECog. The second LV shows younger individuals with higher SCD scores on three CCI questions correlate with lower WMH burden in the temporal and parietal lobes and lower entorhinal cortex volumes. A PCA memory component positively correlated with the second brain LV, suggesting that lower WMH burden may predict better memory performance.

**Conclusions:**

Our findings identified two key LVs that demonstrate that the CCI not only more sensitively detects overall neural decline but may be more sensitive to different etiologies than the ECog.